# Combined analysis of multiple linear regression and principal components for predicting performance indicators in broiler chickens under commercial conditions

**DOI:** 10.1016/j.psj.2025.105728

**Published:** 2025-08-25

**Authors:** Roberto C. Freitas, Arele A. Calderano, Carlos H. Oliveira, Manoel G. Neto, Jansller L. Genova

**Affiliations:** a Department of Animal Science, São Paulo State University, Araçatuba, SP 16050-680, Brazil; b Department of Animal Science, Federal University of Viçosa, Viçosa, MG 36570-900, Brazil

**Keywords:** Broiler farming, Broiler performance, Multiple regression, Principal component, Statistical modeling

## Abstract

This study aimed to assess the influence of biological, environmental, and management factors on broiler performance traits under commercial conditions using multiple linear regression and principal component analysis (PCA). We used a database containing 4,842 records of commercial broiler flocks (Ross 95 and Cobb 500) collected between 2019 and 2022, which included traits such as breeder ages, sex of flocks, type of housing (conventional or climate-controlled houses), stocking densities, placement seasons, litter reutilization, and slaughter ages. Performance responses included body weight (BW) and feed intake at different ages, daily weight gain (DWG), feed conversion ratio (FCR), and production efficiency index (PEI). Predictors were selected by stepwise backward regression using the Akaike information criterion. Multicollinearity was assessed using variance inflation factors, and model was validated using 10-fold cross-validation. The PCA was applied to identify performance patterns across categorical groupings and components retained by Kaiser criterion with explained variance ≥ 60%. Males showed increases of 0.352 kg in BW at 42 days and 27.6 points in PEI compared to females, while broilers from young breeders reduced these values by 0.030 kg and 5.6 points, respectively. Each additional bird/m² increased BW by 0.050 kg and PEI by 7.6 points. Slaughter after 42 days decreased PEI by 5.4 points per day. Summer placements increased BW by 0.107 kg and PEI by 25.2 points compared to winter, while conventional houses in spring and summer led to performance reductions. Feed intake and DWG were influenced by sex of flocks, breeder age, stocking density, and their interactions with season and type of housing. The PCA explained 70.2% of the total variance, with PC1 strongly associated with BW, DWG, and PEI, and negatively with FCR. In conclusion, male and mixed-sex flocks showed superior performance, while broilers from young and second-cycle breeders had lower performance. Intermediate stocking densities (12–14 birds/m²) and earlier slaughter age improved outcomes. Fall and summer placements enhanced performance, although conventional housing under these conditions was detrimental.

## Introduction

The intensification of broiler production systems has led to significant advances in performance, management, and housing, enabling the industry to meet the growing global demand for animal protein. However, this intensification has also increased management complexity and the challenge of identifying key factors affecting performance under commercial conditions, given the variability in genetics (Fernandes et al., 2013), environment (Ferreira et al., 2024), and management practices (Xin et al., 2011) in different production systems.

Several factors have been reported as determinants influencing broiler performance, including bird sex, breeder age, stocking density, season, type of housing, and environmental conditions (Fernandes et al., 2013; Damasceno et al., 2017; Ferreira et al., 2024). Biological factors such as sex directly affect carcass and performance traits (e.g., growth rate and feed efficiency) (Fernandes et al., 2013), while breeder age influences hatchability and embryonic mortality, with subsequent effects on chick quality (Almeida et al., 2008; Ulmer-Franco et al., 2010). Environmental and structural factors, such as stocking density (Dozier et al., 2005; Gholami et al., 2020; Sanchez-Casanova et al., 2021), type of housing (Ferreira et al., 2024), and overall environmental conditions (Gholami et al., 2020; Sanchez-Casanova et al., 2021), affect thermal comfort and bird health, which may limit broiler productive potential.

Despite substantial advances in broiler research, particularly in nutrition, environment, and management, a comprehensive understanding of how multiple factors interact to influence performance under commercial conditions is still limited. Most studies evaluate individual factors in isolation, which limits the accuracy of estimating their effects and reduces the applicability of the results for decision-making under commercial conditions. However, by systematizing performance data and applying multivariate statistical methods (Pinto et al., 2006; Bila and Tyasi, 2022) in combination with predictive modeling techniques (Ahmadi et al., 2008; Yakubu et al., 2009), it has become possible to investigate complex interactions and simultaneously quantify the effects of multiple factors with greater robustness and precision (Pierozan et al., 2016; Borges et al., 2018; Oliveira et al., 2024). For instance, Bila and Tyasi (2022) applied principal component analysis (PCA) to morphological traits in Ross 308 broilers, extracting components that explained up to 57% of the total variance and using them in regression models to predict body weight more accurately than with individual variables. Similarly, Li et al. (2024) used PCA to identify latent environmental factors (e.g., air velocity and humidity) in broiler houses based on sensor data, demonstrating the method's effectiveness in analyzing complex commercial systems.

In particular, few field studies have applied multivariate analyses to commercial datasets, which limits the translation of experimental findings into practical poultry production systems. Therefore, this study aimed to assess the influence of biological, environmental, and management factors on broiler performance traits under commercial conditions using multiple linear regression (e.g., prediction models) and PCA.

## Materials and methods

### *Data collection and variables assessed*

The study collected data from 4,842 commercial broiler flocks between 2019 and 2022 in southeastern Brazil (state of Minas Gerais), using two commercial broiler strains, Ross 308 AP (AP 95) and Cobb 500. Ross 308 AP (AP 95) and Cobb 500 are strains from Aviagen and Cobb-Vantress, respectively, and are commonly used by Brazilian integrators due to their rapid growth, efficient feed conversion, and favorable carcass yield. The dataset comprised 12 dependent and 7 independent variables, obtained under production conditions without invasive procedures or experimental interventions. Management and husbandry practices followed commercial guidelines (Cobb-Vantress, 2021; Aviagen, 2025) and were consistent with the recommendations of the Guide for the Care and Use of Agricultural Animals in Research and Teaching (FASS, 2020).

The independent variables considered were breeder age (young 25–35 weeks, peak-phase 36–45 weeks, post-peak 46–55 weeks, old 56–68 weeks, and second-cycle 69–95 weeks), sex of flocks (male, female, or mixed), type of housing (conventional houses with positive pressure or climate-controlled houses with negative pressure), placement season (spring, summer, fall, or winter), stocking density (birds/m²), litter reuse (reused or non-reused), and slaughter age (up to 42 days, 42-47 days, and above 47 days). The dependent variables included body weight (**BW**, kg) at different ages (7, 14, 21, 28, 35, and 42 days), BW at slaughter, daily weight gain (**DWG, g/day**), feed intake (**FI**, kg/bird) during specific rearing phases (22 to 28 days and 29 days to slaughter). Starter feed intake (1 to 21 days) was excluded from regression and PCA analyses, as preliminary models indicated negligible explanatory power.

For measuring BW, approximately 100 birds per flock (representing 0.5–1.0% of the total flock) were randomly selected from different locations throughout each house to ensure representative sampling and accurate estimates. Weighting was performed manually using calibrated scales. The BW data were recorded on flock control spreadsheets and sent to the company’s management system, enabling weekly monitoring of flock performance. At the end of each production cycle, all records were verified with the company’s data processing center to ensure consistency, traceability, and compliance with standards. The BW at slaughter corresponded to the average live weight recorded by the slaughterhouse. The DWG was calculated by subtracting the initial BW from the final BW and dividing the result by the number of rearing days, thus representing the average growth rate of the flock throughout the entire production period. The FI was calculated based on the records of the feed supply company at each production phase. The quantity supplied was then divided by the number of birds placed, expressed in kg/bird. The feed conversion ratio (**FCR**, kg/kg) was calculated by dividing the total FI by BW gain, and the production efficiency index (**PEI**) was calculated according to Equation 1:

(1) PEI=[(BW×viability)/(age×FCR)]×100,

where viability expresses the livability of the flock and is defined as the percentage of birds remaining at the end of the production cycle in relation to the initial number of birds placed.

### *Statistical procedures*

The data were carefully inspected for missing values and coding consistency. Observations with standardized residuals greater than |3|, leverage exceeding 2p/n (where p is the number of model parameters and n is the sample size), or Cook’s distance > 1 were considered potential outliers or influential points (Montgomery et al., 2012). Categorical variables were converted to factors, and continuous variables were assessed in numeric format. Descriptive statistics for the main variables evaluated are presented in Table 1, including mean and standard deviation.

**Table 1:** Descriptive statistics of stocking density, slaughter age, body weight (BW) at placement, 7, 14, 21, 28, 35, 42 days, and at slaughter, *daily weight gain (DWG), feed intake (FI), feed conversion ratio (FCR), and production efficiency index (PEI) evaluated in 4,842 commercial broiler flocks.*

Variable	Mean	Standard deviation
Stocking density, birds/m²	12.19	1.737
Slaughter age, d	46.27	2.501
BW at placement, kg	0.050	0.005
BW at 7 days, kg	0.170	0.023
BW at 14 days, kg	0.440	0.063
BW at 21 days, kg	0.900	0.117
BW at 28 days, kg	1.500	0.167
BW at 35 days, kg	2.120	0.192
BW at 42 days, kg	2.780	0.226
BW at slaughter, kg	3.040	0.241
DWG, g/d	65.68	4.979
FI 22 to 28 days, kg/bird	1.290	0.200
FI 29 days to slaughter, kg/bird	2.730	0.507
FCR, kg/kg	1.800	0.092
PEI	345.2	42.57

Multiple linear regressions were fitted separately for each response variable. Fixed effects included breeder age, sex of flocks, type of housing, placement season, stocking density, litter reuse, and slaughter age. BW at placement was included as a covariate. The following interactions were tested: type of housing × placement season, type of housing × stocking density, and breeder age × BW at placement.

Categorical predictors were dummy-coded (Oliveira et al., 2024), using the following reference levels: peak-phase breeders, female flocks, climate-controlled houses, winter placement, and non-reused litter. Predictor selection was performed using a stepwise backward procedure based on the Akaike information criterion (**AIC**) (Oliveira et al., 2024). To avoid multicollinearity, the variance inflation factor (**VIF** ≤ 10) and the generalized variance inflation factor (**GVIF**), adjusted for multi-level factors, were evaluated (Fox and Monette, 1992).

Predictive robustness was assessed via 10-fold cross-validation, with estimation of the mean squared prediction error (**MSPE**), mean relative error (**MRE**), and Pearson correlation between observed and predicted values (Oliveira et al., 2024). Final model outputs included estimated coefficients, standard errors, P-values, VIF, and R².

For principal component analysis (**PCA**), all independent and response variables were included. Stocking density and slaughter age were categorized based on practical thresholds: (1) stocking density: 9–11, 12–14, and above 14 birds/m²; (2) slaughter age: up to 42, 42–47, and above 47 days. Response variables were standardized (z-scores). The PCA was performed based on categorical groupings of interest, with components retained following the Kaiser criterion (eigenvalue ≥ 1.0) (Kaiser, 1958) and a cumulative explained variance of 60% or greater. To evaluate whether the multivariate profiles differed significantly among categorical groups, the centroid scores of each group were statistically compared using one-way analysis of variance, followed by Tukey’s honest significant difference test for pairwise comparisons. Group separation was visualized in biplots of the first and second principal components (**PC1** and **PC2**), and results were summarized by eigenvalues, explained variance, and factor loadings. All analyses were performed in RStudio (version 2024.12.1+563; R Core Team, 2024) using the statistical packages car, officer, flextable, broom, caret, tidyverse, FactoMineR, factoextra, ggrepel, patchwork, readxl, stringr, grid, dplyr, and ggplot2.

## Results

### *General performance of multiple linear regression models*

For FI during the starter phase, R² values were only 0.021% (1 to 7 days) and 0.068% (8 to 21 days), indicating no meaningful explanatory power; therefore, these data were not included in the multivariate analyses. The reference categories in the models were peak-phase breeders, female flocks, climate-controlled houses, winter placement, and non-reused litter. The coefficients for each category represent the effect relative to these references. The R² values indicated that FCR (R² = 0.530) and PEI (R² = 0.422) were the most explained by the predictive models. Cross-validation metrics, including MSPE and MRE, confirmed the robustness of the models, with MSPE values less than or equal to 0.098 (except for DWG and PEI) and MRE ranging from 2.6% to 10.7%. Pearson correlation coefficients between observed and predicted values ranged from 0.442 to 0.786, supporting the validity of the predictions.

Among the main estimated effects, flock sex was a determining factor. Compared to female flocks, male flocks showed an increase of 0.352 kg in BW at 42 days (*P <* 0.001), 7.5 g/day in DWG (*P <* 0.001), and 27.6 points in PEI (*P <* 0.001). Similarly, mixed-sex flocks exhibited increases of 0.165 kg in BW at 42 days (*P <* 0.001) and 17.5 points in PEI (*P <* 0.001) compared to female flocks.

Breeder age had a significant impact on performance. Broilers from young breeders showed reductions of 0.030 kg in BW at 42 days (*P =* 0.003), 5.6 points in PEI (*P <* 0.001), and 1.1 g/day in DWG (*P <* 0.001) compared to peak-phase breeders. Similarly, broilers from second-cycle breeders showed a slight reduction of 0.016 kg in BW at 42 days (*P =* 0.188), 10.8 points in PEI (*P <* 0.001), and 1.0 g/day in DWG (*P <* 0.001), compared to peak-phase breeders.

Stocking density had a positive effect on broiler performance. For each additional bird per m², BW at 42 days increased by 0.050 kg (*P <* 0.001), PEI by 7.6 points (*P <* 0.001), and DWG by 0.66 g/day (*P <* 0.001), suggesting improved performance up to a certain threshold. In contrast, slaughter age had a negative effect, reducing PEI by 5.4 points (*P <* 0.001) and DWG by 0.15 g/day (*P <* 0.001) for each additional day.

Regarding season, summer placement increased BW at 42 days by 0.107 kg, PEI by 25.2 points (*P <* 0.001), and DWG by 2.4 g/day (*P <* 0.001) compared to winter. Fall and spring placements also improved broiler performance, although to a lesser extent. However, interactions with the type of housing revealed that, in conventional houses, both spring and summer were associated with performance declines. For example, the spring × conventional house interaction reduced BW at 35 days by 0.059 kg (*P <* 0.001), BW at slaughter by 0.109 kg (*P <* 0.001), and PEI by 13.2 points (*P <* 0.001), while the summer × conventional house interaction led to reductions of 0.034 kg in BW at 35 days (*P =* 0.006), 0.080 kg in BW at slaughter (*P <* 0.001), and 10.2 points in PEI (*P <* 0.001).

### *Identity of the multiple linear regression models*

According to the results presented in Table 2, two significant predictors for FI from 22 to 28 days were male (β = 0.544) and mixed-sex flocks (β = 0.503), indicating higher FI compared to females (*P <* 0.001). Regarding breeder age, an increase in FI was observed only in broilers from old breeders (β = 0.021; *P =* 0.020), whereas those from young (β = –0.028; *P <* 0.001) and second-cycle (β = –0.020; *P =*0.009) breeders showed lower FI compared to those from peak-phase breeders. Post-peak breeders did not differ in FI from the reference (*P =* 0.107). Stocking density (β = –0.010; *P =* 0.009) and slaughter age (β = –0.016; *P <* 0.001) reduced FI. Body weight at placement was positively associated with FI (β = 2.666; *P <* 0.001). Regarding seasons, fall (β = 0.054), spring (β = 0.114), and summer (β = 0.134) increased FI compared to winter (*P <* 0.001). There was a significant interaction between winter placement and conventional houses (β = 0.044; *P =* 0.003), indicating that broilers housed under these conditions consumed approximately 0.044 kg more than those in climate-controlled houses during the same season. Similarly, the fall × conventional house interaction (β = 0.042; *P =* 0.006) also resulted in higher FI. Interactions between spring or summer and conventional houses were not significant (*P >* 0.05).

**Table 2:** Multiple linear regression model for feed intake (FI, kg/bird) from 22 to 28 days in broilers as a function of breeder age, sex of flocks, environment housing, placement season, stocking density (birds/m²), litter reuse, slaughter age (days), and interactions.

Term¹	Estimate	SE	P-value	VIF	R²
Intercept	1.466	0.087	<0.001		0.202
Post-peak breeders	0.013	0.008	0.107		
Young breeders	-0.028	0.007	<0.001		
Second-cycle breeders	-0.020	0.009	0.030		
Old breeders	0.021	0.009	0.020		
Male flocks	0.544	0.031	<0.001		
Mixed-sex flocks	0.503	0.022	<0.001		
Fall placement	0.054	0.010	<0.001		
Spring placement	0.114	0.010	<0.001		
Summer placement	0.134	0.010	<0.001		
Stocking density	-0.010	0.004	0.009	2.456	
Slaughter age	-0.016	0.001	<0.001	1.124	
Body weight at placement	2.666	0.627	<0.001	1.224	
Winter × conventional houses	0.044	0.015	0.003		
Fall × conventional houses	0.042	0.015	0.006		
Spring × conventional houses	-0.002	0.016	0.919		
Summer × conventional houses	-0.005	0.015	0.757		

¹ Body weight at placement was used as a covariate; reference categories: peak-phase breeders, female flocks, climate-controlled houses, winter placement, and non-reused litter; SE = standard error; P-value = probability of significance; VIF = variance inflation factor; R² = coefficient of determination; k-fold MSPE (mean): 0.031; k-fold mean relative error: 0.107; k-fold Pearson correlation: 0.442.

As presented in Table 3, broilers from young breeders showed the most significant reduction in FI from 29 days to slaughter (β = –0.078; *P <* 0.001) compared to those from peak-phase breeders. Broilers from second-cycle breeders (β = –0.040; *P =* 0.012) also showed reduced FI. There was no difference in FI between male and female flocks (*P =* 0.353), whereas mixed-sex flocks showed lower FI (β = –0.143; *P <* 0.001) than female flocks. Conventional houses (β = –0.096; *P <* 0.001) and spring placement (β = –0.051; *P =* 0.005) also reduced FI. In contrast, stocking density (β = 0.053; *P <* 0.001), slaughter age (β = 0.177; *P <* 0.001), and BW at placement (β = 3.292; *P =* 0.003) increased FI. Significant interactions were observed between spring (β = –0.088; *P =* 0.001) or summer (β = –0.053; *P =* 0.036) placement and conventional houses, indicating reduced FI under these conditions. No interaction was observed for fall placement and conventional houses (*P =* 0.178).

**Table 3:** Multiple linear regression model for feed intake (FI, kg/bird) from 29 days to slaughter in broilers as a function of breeder age, sex of flocks, environment housing, placement season, stocking density (birds/m²), litter reuse, slaughter age (days), and interactions.

Term¹	Estimate	SE	P-value	VIF	R²
Intercept	-6.051	0.153	<0.001		0.620
Post-peak breeders	0.020	0.015	0.168		
Young breeders	-0.078	0.013	<0.001		
Second-cycle breeders	-0.040	0.016	0.012		
Old breeders	0.006	0.016	0.699		
Male flocks	0.051	0.055	0.353		
Mixed-sex flocks	-0.143	0.038	<0.001		
Conventional houses	-0.096	0.027	<0.001		
Fall placement	0.185	0.017	<0.001		
Spring placement	-0.051	0.018	0.005		
Summer placement	-0.010	0.017	0.574		
Stocking density	0.053	0.006	<0.001	2.456	
Slaughter age	0.177	0.002	<0.001	1.124	
Body weight at placement	3.292	1.109	0.003	1.224	
Fall × conventional houses	-0.034	0.025	0.178		
Spring × conventional houses	-0.088	0.027	0.001		
Summer × conventional houses	-0.053	0.025	0.036		

¹ Body weight at placement was used as a covariate; reference categories: peak-phase breeders, female flocks, climate-controlled houses, winter placement, and non-reused litter; SE = standard error; P-value = probability of significance; VIF = variance inflation factor; R² = coefficient of determination; k-fold MSPE (mean): 0.098; k-fold mean relative error: 0.099; k-fold Pearson correlation: 0.786.

The multiple linear regression for BW at 7, 14, 21, 28, 35, and 42 days, as well as at slaughter, is presented in Table 4, Table 5, Table 6, Table 7, Table 8, Table 9, Table 10. Male flocks showed higher BW compared to female flocks at 21 (β = 0.043), 28 (β = 0.130), 35 (β = 0.214), 42 (β = 0.352), and at slaughter (β = 0.354) (*P <* 0.010). Similarly, mixed-sex flocks also showed higher BW than females at 21 (β = 0.027), 28 (β = 0.084), 35 (β = 0.109), 42 (β = 0.165), and at slaughter (β = 0.201) (*P <* 0.001). Regarding breeder age, broilers from younger breeders resulted in lower broiler’s BW compared to those from peak-phase breeders at 7 (β = –0.010), 14 (β = –0.021), 21 (β = –0.030), 28 (β = –0.040), 35 (β = –0.038), and 42 days (β = –0.030), and at slaughter (β = –0.051) (*P <* 0.010). Interestingly, only the BW at 7 days of broilers from post-peak (β = 0.003) and old breeders (β = 0.004) was positively influenced (*P <* 0.010), whereas at later ages (14, 21, 28, 35, and 42 days, and at slaughter), the BW of broilers from these breeders did not differ from that of broilers from peak-phase breeders (*P >* 0.05). In contrast, second-cycle breeders did not affect the BW of broilers at 7 and 42 days (*P >* 0.05) but negatively influenced BW at 14 (β = –0.006), 21 (β = –0.019), 28 (β = –0.027), 35 days (β = –0.033), and at slaughter (β = –0.043) (*P ≤* 0.020). Overall, stocking density had positive effects on BW at all evaluated ages (β = 0.005, 0.013, 0.023, 0.032, 0.037, 0.050, and 0.031 at 7, 14, 21, 28, 35, and 42 days, and at slaughter, respectively; *P <* 0.001). In contrast, slaughter age had negative effects on BW at 7 (β = –0.002), 14 (β = –0.006), 21 (β = –0.013), 28 (β = –0.020), 35 (β = –0.029), and 42 days (β = –0.027) (*P <* 0.001). However, it had a positive effect on BW at slaughter (β = 0.059; *P <* 0.001). Regarding litter reutilization, reused litter positively affected BW only at 7 days (β = 0.004; *P =* 0.018). The use of conventional houses positively influenced BW at 14 (β = 0.028), 21 (β = 0.057), 28 (β = 0.052), and 35 days (β = 0.048) (*P <* 0.001), whereas it negatively influenced BW at slaughter (β = –0.043; *P =* 0.008). Additionally, placement season demonstrated that fall, spring, and summer placements had positive effects on BW at 7 (β = 0.015, 0.006, 0.015), 14 (β = 0.048, 0.032, 0.062), 21 (β = 0.099, 0.098, 0.141), 28 (β = 0.130, 0.117, 0.163), 35 (β = 0.112, 0.106, 0.136), and 42 days (β = 0.157, 0.059, 0.107), as well as at slaughter (β = 0.157, 0.072, 0.108) (*P <* 0.001). For interaction effects, the stocking density × conventional houses interaction (β = 0.001; *P <* 0.001) indicated that, for each additional bird per m², BW at 7 days was higher in conventional houses than in climate-controlled. Furthermore, the spring × conventional houses interaction reduced BW at 21 (β = –0.036; *P <* 0.001), 28 (β = –0.033; *P =* 0.005), 35 days (β = –0.059; *P <* 0.001), and at slaughter (β = –0.080; *P <* 0.001), whereas the summer × conventional houses interaction reduced BW at 21 (β = –0.024; *P =* 0.002), 35 days (β = –0.034; *P =* 0.012), and at slaughter (β = –0.080; *P <* 0.001). These interactions suggest that broilers raised in conventional houses had lower BW compared to those in climate-controlled houses during these seasons.

**Table 4:** Multiple linear regression model for body weight at 7 days (kg) in broilers as a function of breeder age, sex of flocks, environment housing, placement season, stocking density (birds/m²), litter reuse, slaughter age (days), and interactions.

Term¹	Estimate	SE	P-value	VIF	R²
Intercept	0.166	0.009	<0.001		0.276
Post-peak breeders	0.003	0.001	0.001		
Young breeders	-0.010	0.001	<0.001		
Second-cycle breeders	-0.001	0.001	0.205		
Old breeders	0.004	0.001	<0.001		
Fall placement	0.015	0.001	<0.001		
Spring placement	0.006	0.001	<0.001		
Summer placement	0.015	0.001	<0.001		
Stocking density	0.005	<0.001	<0.001	2.133	
Reused litter	0.004	0.002	0.018		
Slaughter age	-0.002	<0.001	<0.001	1.120	
Body weight at placement	0.433	0.069	<0.001	1.223	
Stocking density × conventional houses	0.001	<0.001	<0.001		

¹ Body weight at placement was used as a covariate; reference categories: peak-phase breeders, female flocks, climate-controlled houses, winter placement, and non-reused litter; SE = standard error; P-value = probability of significance; VIF = variance inflation factor; R² = coefficient of determination; k-fold MSPE (mean): <0.001; k-fold mean relative error: 0.095; k-fold Pearson correlation: 0.521.

**Table 5:** Multiple linear regression model for body weight at 14 days (kg) in broilers as a function of breeder age, sex of flocks, environment housing, placement season, stocking density (birds/m²), litter reuse, slaughter age (days), and interactions.

Term¹	Estimate	SE	P-value	VIF	R²
Intercept	0.494	0.025	<0.001		0.302
Post-peak breeders	0.004	0.003	0.075		
Young breeders	-0.021	0.002	<0.001		
Second-cycle breeders	-0.006	0.003	0.020		
Old breeders	0.005	0.003	0.070		
Conventional houses	0.028	0.004	<0.001		
Fall placement	0.048	0.002	<0.001		
Spring placement	0.032	0.002	<0.001		
Summer placement	0.062	0.002	<0.001		
Stocking density	0.013	0.001	<0.001	2.453	
Reused litter	0.008	0.004	0.060		
Slaughter age	-0.006	<0.001	<0.001	1.120	
Body weight at placement	0.998	0.188	<0.001	1.223	

¹ Body weight at placement was used as a covariate; reference categories: peak-phase breeders, female flocks, climate-controlled houses, winter placement, and non-reused litter; SE = standard error; P-value = probability of significance; VIF = variance inflation factor; R² = coefficient of determination; k-fold MSPE (mean): 0.003; k-fold mean relative error: 0.100; k-fold Pearson correlation: 0.545.

**Table 6:** Multiple linear regression model for body weight at 21 days (kg) in broilers as a function of breeder age, sex of flocks, environment housing, placement season, stocking density (birds/m²), litter reuse, slaughter age (days), and interactions.

Term¹	Estimate	SE	P-value	VIF	R²
Intercept	1.006	0.047	<0.001		0.341
Post-peak breeders	0.002	0.004	0.624		
Young breeders	-0.030	0.004	<0.001		
Second-cycle breeders	-0.019	0.005	<0.001		
Old breeders	0.004	0.005	0.381		
Male flocks	0.043	0.017	0.010		
Mixed-sex flocks	0.027	0.012	0.021		
Conventional houses	0.057	0.008	<0.001		
Fall placement	0.099	0.005	<0.001		
Spring placement	0.098	0.006	<0.001		
Summer placement	0.141	0.005	<0.001		
Stocking density	0.023	0.002	<0.001	2.453	
Slaughter age	-0.013	0.001	<0.001	1.123	
Body weight at placement	1.622	0.337	<0.001	1.224	
Fall × conventional houses	-0.011	0.008	0.161		
Spring × conventional houses	-0.036	0.008	<0.001		
Summer × conventional houses	-0.024	0.008	0.002		

¹ Body weight at placement was used as a covariate; reference categories: peak-phase breeders, female flocks, climate-controlled houses, winter placement, and non-reused litter; SE = standard error; P-value = probability of significance; VIF = variance inflation factor; R² = coefficient of determination; k-fold MSPE (mean): 0.009; k-fold mean relative error: 0.087; k-fold Pearson correlation: 0.579.

**Table 7:** Multiple linear regression model for body weight at 28 days (kg) in broilers as a function of breeder age, sex of flocks, environment housing, placement season, stocking density (birds/m²), litter reuse, slaughter age (days), and interactions.

Term¹	Estimate	SE	P-value	VIF	R²
Intercept	1.758	0.067	<0.001		0.340
Post-peak breeders	0.004	0.006	0.581		
Young breeders	-0.040	0.006	<0.001		
Second-cycle breeders	-0.027	0.007	<0.001		
Old breeders	0.002	0.007	0.798		
Male flocks	0.130	0.024	<0.001		
Mixed-sex flocks	0.084	0.017	<0.001		
Conventional houses	0.052	0.012	<0.001		
Fall placement	0.130	0.007	<0.001		
Spring placement	0.117	0.008	<0.001		
Summer placement	0.163	0.007	<0.001		
Stocking density	0.032	0.003	<0.001	2.464	
Slaughter age	-0.020	0.001	<0.001	1.123	
Body weight at placement	1.704	0.482	0.001	1.223	
Fall × conventional houses	-0.006	0.011	0.576		
Spring × conventional houses	-0.033	0.012	0.005		
Summer × conventional houses	-0.019	0.011	0.087		

¹ Body weight at placement was used as a covariate; reference categories: peak-phase breeders, female flocks, climate-controlled houses, winter placement, and non-reused litter; SE = standard error; P-value = probability of significance; VIF = variance inflation factor; R² = coefficient of determination; k-fold MSPE (mean): 0.018; k-fold mean relative error: 0.072; k-fold Pearson correlation: 0.582.

**Table 8:** Multiple linear regression model for body weight at 35 days (kg) in broilers as a function of breeder age, sex of flocks, environment housing, placement season, stocking density (birds/m²), litter reuse, slaughter age (days), and interactions.

Term¹	Estimate	SE	P-value	VIF	R²
Intercept	2.704	0.075	<0.001		0.377
Post-peak breeders	0.003	0.007	0.728		
Young breeders	-0.038	0.006	<0.001		
Second-cycle breeders	-0.033	0.008	<0.001		
Old breeders	0.002	0.008	0.826		
Male flocks	0.214	0.027	<0.001		
Mixed-sex flocks	0.109	0.019	<0.001		
Conventional houses	0.048	0.013	<0.001		
Fall placement	0.112	0.008	<0.001		
Spring placement	0.106	0.009	<0.001		
Summer placement	0.136	0.008	<0.001		
Stocking density	0.037	0.003	<0.001	2.454	
Slaughter age	-0.029	0.001	<0.001	1.126	
Body weight at placement	2.163	0.548	<0.001	1.227	
Fall × conventional houses	-0.014	0.013	0.256		
Spring × conventional houses	-0.059	0.013	<0.001		
Summer × conventional houses	-0.034	0.012	0.006		

¹ Body weight at placement was used as a covariate; reference categories: peak-phase breeders, female flocks, climate-controlled houses, winter placement, and non-reused litter; SE = standard error; P-value = probability of significance; VIF = variance inflation factor; R² = coefficient of determination; k-fold MSPE (mean): 0.023; k-fold mean relative error: 0.058; k-fold Pearson correlation: 0.611.

**Table 9:** Multiple linear regression model for body weight at 42 days (kg) in broilers as a function of breeder age, sex of flocks, environment housing, placement season, stocking density (birds/m²), litter reuse, slaughter age (days), and interactions.

Term¹	Estimate	SE	P-value	VIF	R²
Intercept	3.127	0.121	<0.001		0.369
Post-peak breeders	0.014	0.012	0.223		
Young breeders	-0.030	0.010	0.003		
Second-cycle breeders	-0.016	0.012	0.188		
Old breeders	-0.010	0.012	0.434		
Male flocks	0.352	0.045	<0.001		
Mixed-sex flocks	0.165	0.029	<0.001		
Fall placement	0.157	0.014	<0.001		
Spring placement	0.059	0.015	<0.001		
Summer placement	0.107	0.013	<0.001		
Stocking density	0.050	0.005	<0.001	2.380	
Slaughter age	-0.027	0.002	<0.001	1.091	
Body weight at placement	2.115	0.764	0.006	1.182	
Winter × conventional houses	0.042	0.020	0.039		
Fall × conventional houses	-0.008	0.021	0.720		
Spring × conventional houses	-0.008	0.021	0.704		
Summer × conventional houses	-0.010	0.021	0.636		

¹ Body weight at placement was used as a covariate; reference categories: peak-phase breeders, female flocks, climate-controlled houses, winter placement, and non-reused litter; SE = standard error; P-value = probability of significance; VIF = variance inflation factor; R² = coefficient of determination; k-fold MSPE (mean): 0.032; k-fold mean relative error: 0.051; k-fold Pearson correlation: 0.600.

**Table 10:** Multiple linear regression model for body weight at slaughter (kg) in broilers as a function of breeder age, sex of flocks, environment housing, placement season, stocking density (birds/m²), litter reuse, slaughter age (days), and interactions.

Term¹	Estimate	SE	P-value	VIF	R²
Intercept	-0.416	0.094	<0.001		0.380
Post-peak breeders	0.016	0.009	0.063		
Young breeders	-0.051	0.008	<0.001		
Second-cycle breeders	-0.043	0.010	<0.001		
Old breeders	-0.002	0.010	0.869		
Male flocks	0.354	0.034	<0.001		
Mixed-sex flocks	0.201	0.023	<0.001		
Conventional houses	-0.043	0.016	0.008		
Fall placement	0.157	0.010	<0.001		
Spring placement	0.072	0.011	<0.001		
Summer placement	0.108	0.010	<0.001		
Stocking density	0.031	0.004	<0.001	2.456	
Slaughter age	0.059	0.001	<0.001	1.124	
Body weight at placement	3.245	0.676	<0.001	1.224	
Fall × conventional houses	-0.030	0.016	0.054		
Spring × conventional houses	-0.109	0.016	<0.001		
Summer × conventional houses	-0.080	0.015	<0.001		

¹ Body weight at placement was used as a covariate; reference categories: peak-phase breeders, female flocks, climate-controlled houses, winter placement, and non-reused litter; SE = standard error; P-value = probability of significance; VIF = variance inflation factor; R² = coefficient of determination; k-fold MSPE (mean): 0.036; k-fold mean relative error: 0.051; k-fold Pearson correlation: 0.613.

As presented in Table 11, the predictors that positively affected DWG were BW at placement (β = 69.070), male flocks (β = 7.518), mixed-sex flocks (β = 4.225), stocking density (β = 0.663), and seasonal placement, including fall (β = 3.476), spring (β = 1.570), and summer (β = 2.412) (*P <* 0.001). In contrast, broilers from young (β = –1.101) and second-cycle breeders (β = –0.975) showed reduced DWG (*P <* 0.001). Slaughter age also had a negative effect (β = –0.147; *P <* 0.001). Additionally, negative interactions were observed between fall (β = –0.792; *P =* 0.017), spring (β = –2.313; *P <* 0.001), or summer (β = –1.736; *P <* 0.001) placements and conventional houses, indicating reduced DWG in conventional compared to climate-controlled houses during these seasons.

**Table 11:** Multiple linear regression model for daily weight gain (DWG, g/day) in broilers as a function of breeder age, sex of flocks, environment housing, placement season, stocking density (birds/m²), litter reuse, slaughter age (days), and interactions.

Term¹	Estimate	SE	P-value	VIF	R²
Intercept	56.580	2.015	<0.001		0.324
Post-peak breeders	0.332	0.190	0.080		
Young breeders	-1.101	0.169	<0.001		
Second-cycle breeders	-0.975	0.209	<0.001		
Old breeders	-0.024	0.206	0.906		
Male flocks	7.518	0.722	<0.001		
Mixed-sex flocks	4.225	0.499	<0.001		
Conventional houses	-0.929	0.349	0.008		
Fall placement	3.476	0.221	<0.001		
Spring placement	1.570	0.241	<0.001		
Summer placement	2.412	0.221	<0.001		
Stocking density	0.663	0.084	<0.001	2.456	
Slaughter age	-0.147	0.027	<0.001	1.124	
Body weight at placement	69.070	14.576	<0.001	1.224	
Fall × conventional houses	-0.792	0.333	0.017		
Spring × conventional houses	-2.313	0.353	<0.001		
Summer × conventional houses	-1.736	0.330	<0.001		

¹ Body weight at placement was used as a covariate; reference categories: peak-phase breeders, female flocks, climate-controlled houses, winter placement, and non-reused litter; SE = standard error; P-value = probability of significance; VIF = variance inflation factor; R² = coefficient of determination; k-fold MSPE (mean): 16.859; k-fold mean relative error: 0.051; k-fold Pearson correlation: 0.565.

According to the results in Table 12, FCR of broilers was affected by second-cycle breeders (β = 0.014), stocking density (β = –0.011), slaughter age (β = 0.021), and seasonal placement, including fall (β = –0.016), spring (β = –0.040), and summer (β = –0.033) (*P <* 0.001). All interactions between seasonal placement (winter, fall, spring, and summer) and conventional houses were positive (*P ≤* 0.039), indicating higher FCR in conventional houses compared to climate-controlled houses during these seasons.

**Table 12:** Multiple linear regression model for feed conversion ratio (FCR, kg/kg) in broilers as a function of breeder age, sex of flocks, environment housing, placement season, stocking density (birds/m²), litter reuse, slaughter age (days), and interactions.

Term¹	Estimate	SE	P-value	VIF	R²
Intercept	0.955	0.030	<0.001		0.530
Post-peak breeders	-0.002	0.003	0.407		
Young breeders	-0.004	0.003	0.094		
Second-cycle breeders	0.014	0.003	<0.001		
Old breeders	0.004	0.003	0.213		
Fall placement	-0.016	0.003	<0.001		
Spring placement	-0.040	0.004	<0.001		
Summer placement	-0.033	0.003	<0.001		
Stocking density	-0.011	0.001	<0.001	2.453	
Slaughter age	0.021	0.001	<0.001	1.121	
Body weight at placement	0.337	0.222	0.129	1.224	
Winter × conventional houses	0.014	0.005	0.009		
Fall × conventional houses	0.011	0.005	0.039		
Spring × conventional houses	0.027	0.006	<0.001		
Summer × conventional houses	0.020	0.006	<0.001		

¹ Body weight at placement was used as a covariate; reference categories: peak-phase breeders, female flocks, climate-controlled houses, winter placement, and non-reused litter; SE = standard error; P-value = probability of significance; VIF = variance inflation factor; R² = coefficient of determination; k-fold MSPE (mean): 0.004; k-fold mean relative error: 0.026; k-fold Pearson correlation: 0.727.

The multiple linear regression for PEI is presented in Table 13. Male (β = 27.579) and mixed-sex flocks (β = 17.487) showed higher PEI compared to female flocks (*P <* 0.001). Regarding breeder age, broilers from young (β = –5.639) and second-cycle breeders (β = –10.827) exhibited lower PEI (*P <* 0.001). Additionally, PEI was positively associated with stocking density (β = 7.592) and seasonal placement, including fall (β = 23.478), spring (β = 21.077), and summer (β = 25.228), and negatively associated with slaughter age (β = –5.393) (*P <* 0.001). Significant interactions were found between spring (β = –13.205) or summer (β = –10.228) placements and conventional houses, indicating reduced PEI under these conditions (*P <* 0.001).

**Table 13:** Multiple linear regression model for production efficiency index (PEI) in broilers as a function of breeder age, sex of flocks, environment housing, placement season, stocking density (birds/m²), litter reuse, slaughter age (days), and interactions.

Term¹	Estimate	SE	P-value	VIF	R²
Intercept	465.516	15.842	<0.001		0.422
Post-peak breeders	2.608	1.492	0.081		
Young breeders	-5.639	1.330	<0.001		
Second-cycle breeders	-10.827	1.639	<0.001		
Old breeders	-0.377	1.619	0.816		
Male flocks	27.579	5.677	<0.001		
Mixed-sex flocks	17.487	3.926	<0.001		
Conventional houses	-1.712	2.746	0.533		
Fall placement	23.478	1.741	<0.001		
Spring placement	21.077	1.892	<0.001		
Summer placement	25.228	1.738	<0.001		
Stocking density	7.592	0.659	<0.001	2.456	
Slaughter age	-5.393	0.211	<0.001	1.124	
Body weight at placement	198.046	114.626	0.084	1.224	
Fall × conventional houses	-4.727	2.616	0.071		
Spring × conventional houses	-13.205	2.775	<0.001		
Summer × conventional houses	-10.228	2.595	<0.001		

¹ Body weight at placement was used as a covariate; reference categories: peak-phase breeders, female flocks, climate-controlled houses, winter placement, and non-reused litter; SE = standard error; P-value = probability of significance; VIF = variance inflation factor; R² = coefficient of determination; k-fold MSPE (mean): 1042.757; k-fold mean relative error: 0.077; k-fold Pearson correlation: 0.647.

### *Simulations of the validated multiple linear regression models*

Simulations were performed using the estimated β coefficients from the models to demonstrate the impact of the most relevant variables on broiler performance.

Considering a female standard flock from peak-phase breeders reared in climate-controlled houses, with winter placement, non-reused litter (reference, β = 0), a stocking density of 14 birds/m², a slaughter age of 45 days, and a BW at placement of 0.043 kg, the estimated FI from 22 to 28 days was 0.721 kg as expressed by the following equation:

(2) $$\begin{array}{c} \text{FI}\left(22-28\text{days}\right)=1.466+\left(-0.010\times 14\right)+\left(-0.016\times 45\right)+\left(2.666\times 0.043\right) \end{array}$$

By changing the flock sex to male (β = 0.544), the estimated FI would increase to 1.265 kg. Regarding the seasonal effect, if the flock were placed in summer (β = 0.134), the equation would increase to 0.855 kg.

For FI from 29 days to slaughter, considering broilers from young breeders (β = –0.078), reared in conventional houses (β = –0.096), placed in fall (β = 0.185), with a stocking density of 16 birds/m², a slaughter age of 45 days, and a BW at placement of 0.043 kg, the estimated FI was 2.915 kg as expressed in the following equation:

(3) $$\begin{array}{c} \text{FI}\left(29\text{days}-\text{slaughter}\right)=-6.051-0.078-0.096+0.185+\left(0.053\times 16\right)+\left(0.177\times 45\right)+\left(3.292\times 0.043\right) \end{array}$$

For BW at 7 days, considering broilers from young breeders (β = –0.010), placed in spring (β = 0.006), with a stocking density of 14 birds/m², a slaughter age of 45 days, and a BW at placement of 0.045 kg, the estimated BW was 0.161 kg as expressed in the following equation:

(4) $$\begin{array}{c} \text{BW}\text{at}7\mathrm{days}=0.166-0.010+0.006+\left(0.005\times 14\right)-\left(0.002\times 45\right)+\left(0.433\times 0.045\right) \end{array}$$

For BW at 21 days, using the reference traits (female flock from peak-phase breeders, climate-controlled house, winter placement, non-reused litter), with a stocking density of 14 birds/m², a slaughter age of 45 days, and a BW at placement of 0.045 kg, the estimated BW was 0.816 kg as expressed in the following equation:

(5) $$\begin{array}{c} \text{BW}\text{at}21\text{days}=1.006+\left(0.023\times 14\right)+\left(-0.013\times 45\right)+\left(1.622\times 0.045\right) \end{array}$$

For BW at 42 days, under the same reference conditions, the estimated BW was 2.707 kg as expressed in the following equation:

(6) $$\begin{array}{c} \text{BW}\text{at}42=3.127+\left(0.050\times 14\right)+\left(-0.027\times 45\right)+\left(2.115\times 0.045\right) \end{array}$$

By changing the sex to male (β = 0.109), considering broilers from second-cycle breeders (β = –0.043) and placed in spring (β = 0.059), while maintaining the same density, slaughter age, and BW at placement, the predicted BW at 42 was increase to 3.102 kg as expressed in the following equation:

(7) $$\begin{array}{c} \text{BW}\text{at}42=3.127+0.109-0.043+0.059+\left(0.050\times 14\right)+\left(-0.027\times 45\right)+\left(2.115\times 0.045\right) \end{array}$$

For DWG, considering male flocks (β = 7.518), broilers from young breeders (β = –1.101), placed in summer (β = 2.412), with a stocking density of 16 birds/m², a slaughter age of 45 days, and a BW at placement of 0.045 kg, the estimated DWG was 72.510 g/day as expressed in the following equation:

(8) $$\begin{array}{c} \text{DWG}=56.580-1.101+7.518+2.412+\left(0.663\times 16\right)-\left(0.147\times 45\right)+\left(69.070\times 0.045\right) \end{array}$$

For FCR, considering conventional houses (interaction with spring β = 0.027), spring placement (β = –0.040), and broilers from second-cycle breeders (β = 0.014), the estimated FCR was 0.956 kg/kg as expressed in the following equation:

(9) $$\begin{array}{c} \text{FCR}=0.955+0.014-0.040+0.027 \end{array}$$

For PEI, considering male flocks (β = 27.579), broilers from young breeders (β = –5.639), placed in summer (β = 25.228), with a stocking density of 16 birds/m², a slaughter age of 45 days, and a BW at placement of 0.045 kg, the estimated PEI was 400.383 as expressed in the following equation:

(10) $$\begin{array}{c} \text{PEI}=465.516-5.639+27.579+25.228+\left(7.592\times 16\right)-\left(5.393\times 45\right)+\left(198.046\times 0.045\right) \end{array}$$

### *Principal component analysis*

The global PCA explained 70.2% of the total variance in the dataset using the first two components (PC1 = 55.4% and PC2 = 14.9%), with eigenvalues of 6.6 and 1.8, respectively (Table 14). PC1 showed strong positive correlations with all BW measures at all ages, DWG, and the PEI, with loading values exceeding 0.75. Notably, DWG (r = 0.891), BW at 35 days (r = 0.875), and PEI (r = 0.855) exhibited the strongest associations. In contrast, FCR loaded negatively on PC1 (r = –0.578), indicating an inverse relationship with productive traits. PC2, in turn, was predominantly influenced by the FI from 29 days to slaughter, which exhibited a strong positive loading (r = 0.936), and by BW at slaughter (r = 0.662). The FI from 22 to 28 days showed a moderate negative loading (r = –0.466) to PC2, suggesting a temporal shift in intake patterns. FCR (r = 0.256) and DWG (r = 0.278) had secondary contributions to PC2. Notably, FI from 22 to 28 days showed a weak correlation with PC1 (r = 0.313).

**Table 14:** Eigenvalues, explained and cumulative variance (%), and correlation coefficients (loadings) of the response variables with the first two principal components (PC1 and PC2) from the global principal component analysis.

Item		Principal components	
		PC1	PC2
Eigenvalue		6.6	1.8
Explained variance (%)		55.4	14.9
Cumulative variance (%)		55.4	70.2
Response variable		PC1	PC2
Body weight at 7 days, kg		0.758	-0.096
Body weight at 14 days, kg		0.823	-0.141
Body weight at 21 days, kg		0.839	-0.172
Body weight at 28 days, kg		0.864	-0.159
Body weight at 35 days, kg		0.875	-0.148
Body weight at 42 days, kg		0.845	-0.063
Body weight at slaughter, kg		0.670	0.662
Daily weight gain, g/day		0.891	0.278
Feed conversion ratio, kg/kg		-0.578	0.256
Production efficiency index		0.855	0.044
Feed intake (22 to 28 days), kg/bird		0.313	-0.466
Feed intake (29 days to slaughter), kg/bird		0.234	0.936

**Fig. 1:**
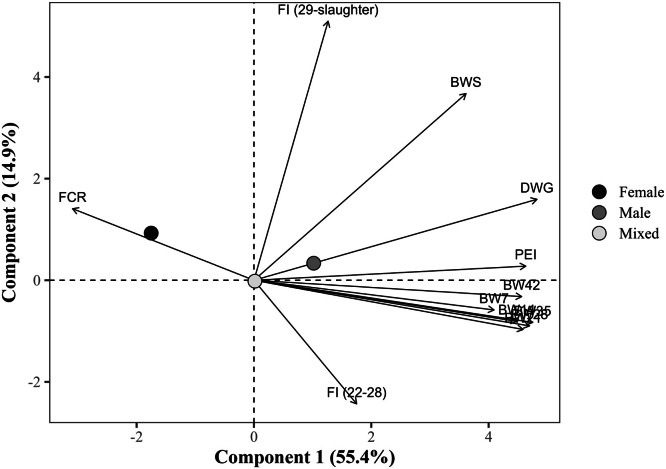
Projection of group means (sex of flocks) in the space of the first two principal components (PC1 and PC2) from the global principal component analysis, with response variable loadings represented as vectors. The points represent the means of the group scores for each principal component. The percentages in parentheses indicate the variance explained by each component. BW7, 14, 21, 28, 35, 42: body weight at 7, 14, 21, 28, 35, and 42 days of age (in kg); BWS: body weight at slaughter (in kg); DWG: daily weight gain (in g); FI (22-28): Feed intake from 22 to 28 days of age (in kg/bird); FI (29 days-slaughter): Feed intake from 29 days of age to slaughter (in kg/bird); FCR: feed conversion ratio (in kg/kg); PEI: production efficiency index.

In the flocks' sex grouping, the vectors for BW (from BW at 7 days to BW at 42 days), DWG, and PEI aligned predominantly along the positive side of PC1, indicating their high positive correlations (Fig. 1). Male flocks clustered in the positive quadrant of PC1 and PC2 (PC1 = +1.17; PC2 = +0.28), indicating superior performance metrics. Female flocks (PC1 = –1.66; PC2 = +1.05) were located in the upper-left quadrant, closer to the FCR vector and opposite to the performance-related variables, reflecting lower performance. Mixed-sex flocks’ group was concentrated near the origin (PC1 ≈ 0), indicating intermediate performance. The centroid for males was significantly (*P <* 0.05) higher in PC1 than that for females (diff = 2.82) and mixed-sex groups (diff = –1.16). The vector of FCR pointed in the opposite direction to BW and PEI, confirming its negative correlation.

**Fig. 2:**
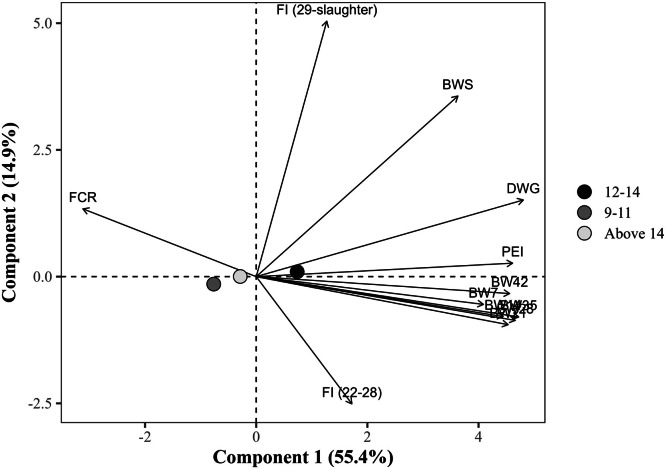
Projection of group means (stocking density, birds/m²) in the space of the first two principal components (PC1 and PC2) from the global principal component analysis, with response variable loadings represented as vectors. The points represent the means of the group scores for each principal component. The percentages in parentheses indicate the variance explained by each component. BW7, 14, 21, 28, 35, 42: body weight at 7, 14, 21, 28, 35, and 42 days of age (in kg); BWS: body weight at slaughter (in kg); DWG: daily weight gain (in g); FI (22-28): Feed intake from 22 to 28 days of age (in kg/bird); FI (29 days-slaughter): Feed intake from 29 days of age to slaughter (in kg/bird); FCR: feed conversion ratio (in kg/kg); PEI: production efficiency index.

Broilers housed at 12–14 birds/m² showed the most favorable multivariate profile, located in the positive direction of PC1 (+0.75), close to performance-related vectors (e.g., BW, PEI, and DWG) (Fig. 2). Low (9–11 birds/m²) and high (>14 birds/m²) stocking densities were associated with greater dispersion or proximity to vectors of reduced performance (e.g., higher FCR). The centroid for the low-density group was in the negative PC1 and PC2 quadrant (–0.76; –0.14), indicating lower performance metrics. Stocking densities above 14 birds/m² were slightly negative on PC1 (–0.31) and slightly closer to FCR, indicating some reduction in performance. The centroids for densities were significantly (*P <* 0.05) different from each other in PC1. Specifically, densities at 12–14 birds/m² differed significantly from low (–1.51) and high (–1.06), while high density differed by 0.45 units compared to low density.

**Fig. 3:**
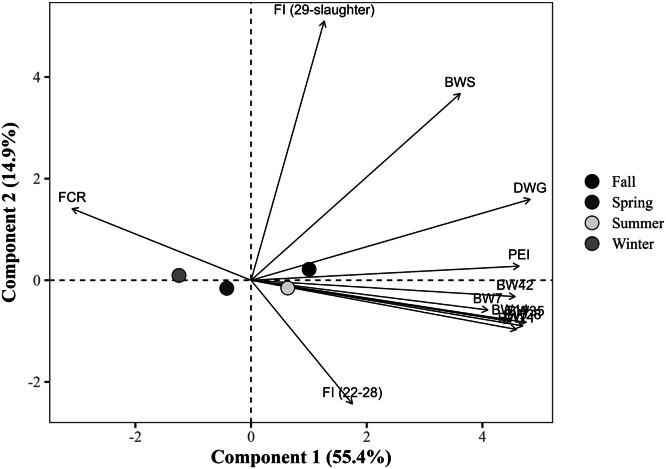
Projection of group means (placement seasons) in the space of the first two principal components (PC1 and PC2) from the global principal component analysis, with response variable loadings represented as vectors. The points represent the means of the group scores for each principal component. The percentages in parentheses indicate the variance explained by each component. BW7, 14, 21, 28, 35, 42: body weight at 7, 14, 21, 28, 35, and 42 days of age (in kg); BWS: body weight at slaughter (in kg); DWG: daily weight gain (in g); FI (22-28): Feed intake from 22 to 28 days of age (in kg/bird); FI (29 days-slaughter): Feed intake from 29 days of age to slaughter (in kg/bird); FCR: feed conversion ratio (in kg/kg); PEI: production efficiency index.

Broilers placed in fall (PC1 = +1.00) were positioned in the quadrant as performance vectors (positive PC1), indicating improved growth performance metrics (Fig. 3). Fall placement birds cluster near the PEI and DWG vectors. The group placed in summer showed moderate positive PC1 (+0.59), but slight negative PC2 (–0.16), pointing to intermediate growth. Birds placed in winter (PC1 = –1.21) and spring (PC1 = –0.46) had negative PC1 values, indicative of reduced performance. Winter aligns slightly with FCR, in the opposite direction of growth performance vectors. The centroids for season were significantly (*P <* 0.05) different from each other in PC1. Birds placed during winter showed markedly lower centroid scores on PC1 compared to those placed in fall (–2.21), spring (–0.75), and summer (–1.80). Additionally, spring placement differed significantly from fall (–1.46) and summer (+1.04), while summer placement also presented lower scores than fall (–0.42).

**Fig. 4:**
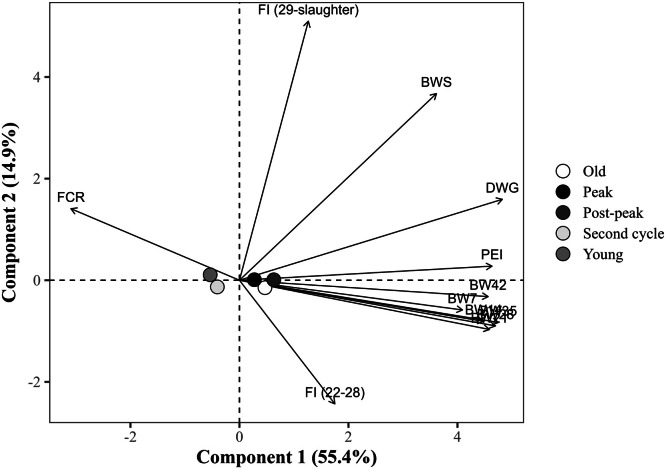
Projection of group means (breeder age) in the space of the first two principal components (PC1 and PC2) from the global principal component analysis, with response variable loadings represented as vectors. The points represent *the means of the group scores for each principal component. The percentages in parentheses indicate the variance explained by each component. BW7, 14, 21, 28, 35, 42: body weight at 7, 14, 21, 28, 35, and 42 days of age (in kg); BWS: body weight at slaughter (in kg); DWG: daily weight gain (in g); FI (22-28): Feed intake from 22 to 28 days of age (in kg/bird); FI (29 days-slaughter): Feed intake from 29 days of age to slaughter (in kg/bird); FCR: feed conversion ratio (in kg/kg); PEI: production efficiency index.*

Broilers from post-peak and old breeders were positively associated with PC1 (0.58 and 0.46, respectively), indicating improved performance metrics (Fig. 4). Broilers from young breeders (PC1 = –0.51; PC2 = +0.12) and second-cycle breeders (–0.42; –0.17) clustered in the negative region of PC1, indicating associations with lower BW and growth rates across the rearing period, and reinforcing their lower performance potential. Broilers from peak-phase breeders (PC1 = +0.29; PC2 = +0.009) were positioned near the origin of the PCA plot, showing an intermediate profile, without strong associations with extremes of performance. Broilers from young and second-cycle breeders showed significantly lower PC1 scores compared to those from old, peak-phase, and post-peak breeders (all *P <* 0.001). Specifically, the young breeder group differed significantly from old (diff = –0.96, *P <* 0.001), peak-phase (diff = –0.80, *P <* 0.001), and post-peak (diff = –1.09, *P <* 0.001). Similarly, the second-cycle group presented significantly lower PC1 scores than old (diff = –0.88, *P <* 0.001), peak-phase (diff = –0.71, *P <* 0.001), and post-peak breeders (diff = –1.00, *P <* 0.001). No significant differences were observed among peak-phase, old, and post-peak breeders (*P >* 0.05) in PC1. The separation along PC1 reflects the contribution of breeder age to offspring performance.

**Fig. 5:**
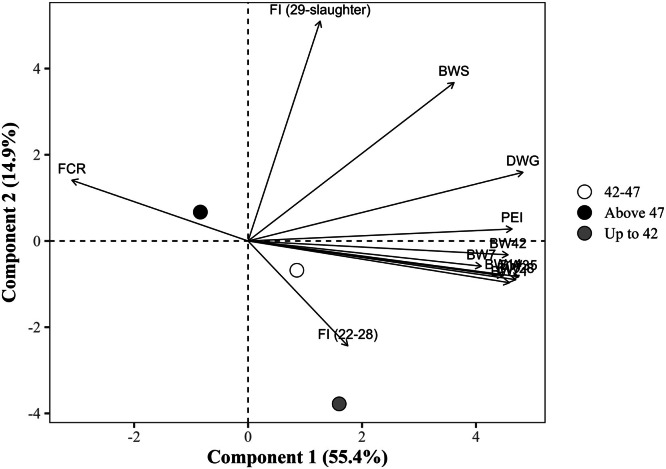
Projection of group means (slaughter age) in the space of the first two principal components (PC1 and PC2) from the global principal component analysis, with response variable loadings represented as vectors. The points represent the means of the group sco*res for each principal component. The percentages in parentheses indicate the variance explained by each component. BW7, 14, 21, 28, 35, 42: body weight at 7, 14, 21, 28, 35, and 42 days of age (in kg); BWS: body weight at slaughter (in kg); DWG: daily weight gain (in g); FI (22-28): Feed intake from 22 to 28 days of age (in kg/bird); FI (29 days-slaughter): Feed intake from 29 days of age to slaughter (in kg/bird); FCR: feed conversion ratio (in kg/kg); PEI: production efficiency index.*

Those slaughtered up to 42 days of age located in the lower-right quadrant had a strongly positive position on PC1 (+1.56) and negative on PC2 (–3.81), indicating high growth rates and efficient FCR (Fig. 5). As slaughter age increased, the centroid values progressively shifted toward negative PC1 and positive PC2. Broilers slaughtered between 42 and 47 days group (PC1 = +0.91; PC2 = –0.67) remained in the positive PC1 region, although closer to the origin than the earlier group. They were still associated with high performance, though less extreme. In contrast, broilers slaughtered after 47 days (PC1 = –0.90; PC2 = +0.67) shifted to the negative PC1 and positive PC2 region. This group was positioned near the vector representing FI from 29 days to slaughter, highlighting an increase in FI during the final stages, possibly leading to a higher FCR. Birds slaughtered after 47 days of age showed significantly lower PC1 scores compared to those slaughtered between 42 and 47 days, with a mean difference of –1.81 units (*P <* 0.001). Although numerically higher, the centroid for up to 42 days did not differ statistically (*P >* 0.05) from either 42 to 47 days or above 47 days.

**Fig. 6:**
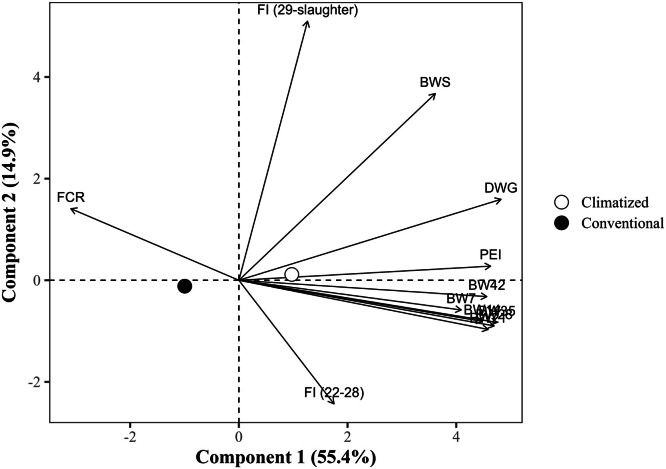
Projection of group means (type of housing) in the space of the first two principal components (PC1 and PC2) from the global principal component analysis, with response variable loadings represented as vectors. The points represent the means of the group scores for each principal component. The percentages in parentheses indicate the variance explained by each component. BW7, 14, 21, 28, 35, 42: body weight at 7, 14, 21, 28, 35, and 42 days of age (in kg); BWS: body weight at slaughter (in kg); DWG: daily weight gain (in g); FI (22-28): Feed intake from 22 to 28 days of age (in kg/bird); FI (29 days-slaughter): Feed intake from 29 days of age to slaughter (in kg/bird); FCR: feed conversion ratio (in kg/kg); PEI: production efficiency index.

The PCA grouped by type of housing revealed separation along PC1, with climate-controlled housing positioned in the performance-oriented quadrant (PC1 = +0.98), associated with higher BW and DWG (Fig. 6). The vectors for BW variables and PEI extended toward the positive PC1 axis, coinciding with the centroid of climate-controlled group. Conversely, conventional housing appeared in the negative direction of PC1 (PC1 = –1.17) and closer to FCR, suggesting less favorable performance. The centroid for climate-controlled housing was significantly (diff = –2.14, *P <* 0.05) higher in PC1 than conventional housing.

**Fig. 7:**
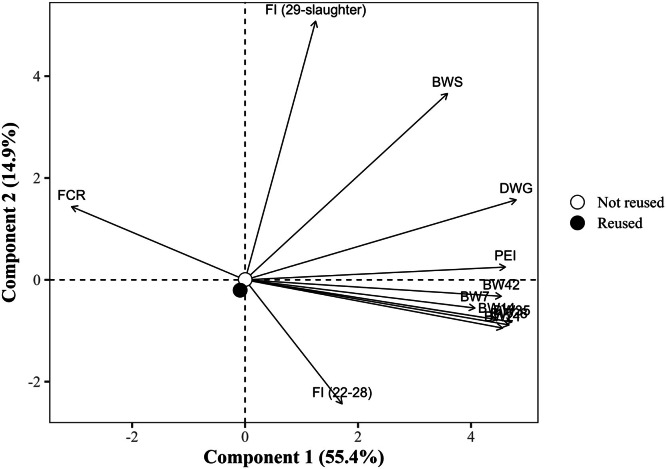
Projection of group means (litter reutilization) in the space of the first two principal components (PC1 and PC2) from the global principal component analysis, with response variable loadings represented as vectors. The points represent the means of the group scores for each principal component. The percentages in parentheses indicate the variance explained by each component. BW7, 14, 21, 28, 35, 42: body weight at 7, 14, 21, 28, 35, and 42 days of age (in kg); BWS: body weight at slaughter (in kg); DWG: daily weight gain (in g); FI (22-28): Feed intake from 22 to 28 days of age (in kg/bird); FI (29 days-slaughter): Feed intake from 29 days of age to slaughter (in kg/bird); FCR: feed conversion ratio (in kg/kg); PEI: production efficiency index.

The PCA based on litter reutilization exhibited overlapping groups, with no difference between the centroids (*P >* 0.05, Fig. 7). The biplot contrasting reused and non-reused litter reveals minimal centroid separation, suggesting subtle overall effects. Birds reared on non-reused litter (PC1 ≈ 0; PC2 ≈ 0) did not show any directional association with performance vectors, indicating more neutral or variable outcomes. However, birds reared on reused litter were shifted to the negative side of both axes (PC1 = –0.15; PC2 = –0.21), indicating an association with reduced performance. The lack of sharp separation may reflect that performance differences are dependent on litter management quality rather than reuse itself.

## Discussion

In the present study, predictive modeling and multivariate analysis were effective tools for identifying critical biological, environmental, and management factors influencing broiler performance under commercial conditions. As reported in previous studies (Pinto et al., 2006; Ahmadi et al., 2008; Yakubu et al., 2009; Bila and Tyasi, 2022), the application of robust statistical models has been essential to understanding the effects of production and environmental factors on key performance indicators, such as DWG, FI, and FCR.

In the current study, factors such as flock sex, breeder age, stocking density, placement season, and housing types showed significant influence on broiler performance and productivity, indicating that their effect is integrated and dependent on environmental and management conditions. Our results demonstrated that male flocks, broilers from peak or post-peak breeders, and fall or summer placements in climate-controlled houses exhibited the best performance indicators. These findings are consistent with previous studies that reported biological (sex, lineage, breeder age) and environmental (thermal environment, season) influences on broiler performance and health (Fernandes et al., 2013; Gholami et al., 2020). These findings reinforce the strategic importance of adopting separate-sex flocking and adjusting management according to breeder age and seasonal variations to enhance flock uniformity and productivity.

The positive influence of stocking density on broiler performance agrees with previous findings (Sanchez-Casanova et al., 2021). Our findings showed that stocking density above 16 birds/m² resulted in increased performance (e.g., BW, PEI, and DWG), although the magnitude of response per additional unit was lower compared to intermediate densities. This result may be attributed to strict climate control, as all flocks housed above 12 birds/m² were reared in climate-controlled houses. This contrasts with other studies (Gholami et al., 2020; Dozier et al., 2006), which reported that higher stocking densities reduced broiler performance due to the absence of strict climate control, resulting in heat stress and competition for resources. Therefore, stocking density should not be considered in isolation; rather, it must be contextualized within the housing environment. High stocking density combined with inadequate facilities may increase the risk of losses due to social and heat stress, as suggested by Bizeray et al. (2002). Therefore, our findings support practical recommendations for adjusting stocking density according to season and housing types, without compromising performance within the evaluated range.

The effectiveness of climate-controlled houses in maintaining birds within the thermoneutral zone may explain the significant improvements in broiler performance, especially during the most thermally challenging seasons (Damasceno et al., 2017), such as summer and spring. Ferreira et al. (2024) highlighted that climate-controlled facilities ensure thermal stability, leading to reduced heat stress and improved performance and flock uniformity. Conversely, conventional houses may accentuate the adverse effects of extreme weather conditions, significantly reducing broiler productivity. Therefore, maintaining thermoneutral conditions and controlling environmental factors is essential, although not directly assessed in the present study, but as demonstrated in studies with laying hens (Xin et al., 2011) and broiler chickens (Damasceno et al., 2017). Furthermore, the observed interaction between housing types and placement season suggests that investments in climate control infrastructure can lead to direct improvements in productivity and economic return under intensive production systems.

The superior performance of male flocks observed in this study, expressed as higher DWG and better efficiency, may be explained by physiological mechanisms involving metabolic and hormonal differences (England et al., 2022) associated with sexual dimorphism, as described by Fernandes et al. (2013). This dimorphism leads to greater muscle deposition capacity and enhanced performance in males, which supports the use of separate-sex flocking under commercial conditions to optimize productivity. When males and females are reared separately, specific management strategies can be applied to further optimize flock performance, such as implementing sex-specific nutritional programs, adjusting stocking density, and targeting different slaughter weights, thereby enhancing flock uniformity and processing efficiency (Cobb-Vantress, 2021; Aviagen, 2025).

Our results showed lower performance in broilers from young or second-cycle breeders, in agreement with previous studies (Almeida et al., 2008; Ulmer-Franco et al., 2010), which reported reduced nutrient transfer to the embryo and higher early embryonic mortality at these breeder ages. This reduction in nutrient supply may compromise one-day-old chick quality and lead to subsequent declines in performance. For example, Ulmer-Franco et al. (2010) observed that chicks from older breeders were heavier and exhibited better early viability.

The PCA supported these findings by clustering groups within quadrants associated with better performance metrics and highlighting the importance of variables such as BW, DWG, and PEI in group separation. The agreement between the results from multiple linear regression and PCA underscores the robustness of the associations identified in this study, as also observed by Bila and Tyasi (2022) and Li et al. (2024) in studies with broilers. Pierozan et al. (2016) and Oliveira et al. (2024) showed that multivariate statistics and cross-validation allow the assessment of the generalizability of predictive models and their application in real animal production systems, highlighting the relevance of the current study to predict and optimize broiler performance under commercial conditions.

Cross-validation confirmed that the model applied in this study had high predictive robustness, as indicated by low MSPE and MRE values, demonstrating reliable predictive capacity and strong generalizability to commercial conditions similar to those assessed in this study. These results are consistent with previous modeling studies in animal production (Borges et al., 2018; Bila and Tyasi, 2022; Oliveira et al., 2024). These metrics, widely used in animal science modeling, confirm that the model captures the variability present in commercial datasets and can support evidence-based management under similar operational conditions. Thus, our findings reinforce the importance of integrating biological, environmental, and management factors to improve prediction accuracy and, consequently, support decision-making in intensive broiler production systems.

In conclusion, male and mixed-sex flocks exhibited superior performance indicators. Broilers from young and second-cycle breeders had lower performance. Increasing stocking density positively influenced performance up to a certain threshold, whereas longer slaughter age was consistently associated with poorer outcomes. Birds placed in fall and summer showed better overall performance. However, significant interactions between housing type and placement season revealed that conventional housing during spring and summer impaired performance. Principal component analysis supported the regression findings, with PC1 explaining the primary variance in performance traits. Males, birds from older or post-peak breeders, intermediate densities (12–14 birds/m²), fall placement, earlier slaughter age, and climate-controlled houses were grouped in the high-performance quadrant, with positive correlations with BW, DWG, and PEI, and negative correlations with FCR. Conversely, birds from young or second-cycle breeders, subjected to extreme densities, longer slaughter ages, conventional housing, and less favorable seasons were associated with reduced performance.

## Funding

This research did not receive any specific grant from funding agencies in the public, commercial, or not-for-profit sectors.

## Disclosures

None of the authors of this paper have a financial or personal relationship with other people or organizations that could inappropriately influence or bias the content of the paper. All authors declare that they have no competing interests. All data generated or analyzed during this study are available from the corresponding author upon reasonable request.

## CRediT authorship contribution statement

**Roberto C. Freitas:** Conceptualization, Data curation, Investigation, Visualization. **Arele A. Calderano:** Conceptualization, Methodology, Visualization, Writing – original draft, Writing – review & editing. **Carlos H. Oliveira:** Visualization, Writing – original draft, Writing – review & editing. **Manoel G. Neto:** Project administration, Supervision, Validation, Visualization. **Jansller L. Genova:** Conceptualization, Data curation, Formal analysis, Supervision, Validation, Visualization, Writing – original draft, Writing – review & editing.
